# Serum Zonulin Measured by Commercial Kit Fails to Correlate With Physiologic Measures of Altered Gut Permeability in First Degree Relatives of Crohn's Disease Patients

**DOI:** 10.3389/fphys.2021.645303

**Published:** 2021-03-25

**Authors:** Namita Power, Williams Turpin, Osvaldo Espin-Garcia, Michelle I. Smith, The CCC GEM Project Research Consortium, Kenneth Croitoru

**Affiliations:** ^1^Zane Cohen Centre for Digestive Diseases, Mount Sinai Hospital, Toronto, ON, Canada; ^2^Department of Gastroenterology, Lunenfeld-Tanenbaum Research Institute, Toronto, ON, Canada; ^3^Department of Medicine, University of Toronto, Toronto, ON, Canada; ^4^Division of Biostatistics, Dalla Lana School of Public Health, University of Toronto, Toronto, ON, Canada

**Keywords:** gut barrier, inflammatory bowel disease, zonulin, lactulose-mannitol ratio test, intestinal fatty acid binding proteins

## Abstract

Intestinal epithelial cell tight junctions (TJs) contribute to the integrity of the intestinal barrier allowing for control of the physical barrier between external antigens or bacterial products and the internal environment. Zonula occludens-1 (ZO-1) is a protein that modulates intestinal TJs, and serum levels of ZO-1 has been suggested as a biomarker of disrupted barrier function in humans. Previous studies suggested that increased intestinal permeability was associated with evidence of TJ abnormalities. However, there is limited information on the serological measurement of ZO-1 and its relation to other tests of barrier function in healthy subjects. We investigated the correlation of serum ZO-1, with physiologic measures of intestinal permeability (as the ratio of the fractional excretion of lactulose-mannitol or LMR) in a cohort of 39 healthy FDRs of Crohn's disease (CD) patients. No significant correlation was found between LMR and ZO-1 levels (*r*2 = 0.004, *P* < 0.71), or intestinal fatty acid binding proteins (I-FABP) (*r*2 = 0.004, *P* < 0.71). In conclusion, our data show that ZO-1 and I-FABP are not a marker of gut permeability as defined by LMR.

## Introduction

Intestinal permeability is one measure of alterations in gut barrier function in humans. It has been associated with multiple pathologies such as Crohn's disease (CD), ulcerative colitis (UC), or celiac disease (Michielan and D'Inca, 2015). Furthermore, there is emerging evidence for the role of impaired intestinal permeability even outside of the gut such as in metabolic diseases including obesity or type-2 diabetes. Studies have shown that abnormal intestinal permeability is a feature of disrupted intestinal homeostasis and its measurement may be used as a tool to assess gastrointestinal function (Harris et al., 2012; Teixeira et al., 2012; Michielan and D'Inca, 2015; Teshima et al., 2017).

Transepithelial passage of molecules across the intestinal epithelium occurs through the transcellular pathway, the paracellular or unrestricted pathway (Zuo et al., 2020). Intestinal epithelial cell tight junctions (TJs) contribute to the integrity of the intestinal barrier allowing for control of the physical barrier between external antigens or bacterial products and the internal environment. TJ are the most apical junctional complex connecting adjacent epithelial cells and controls the paracellular flow molecules through the intestinal epithelia (Turner, 2009). TJ structure can be regulated by many intrinsic and extrinsic factors including cytokines, growth factors, cellular stress, pathogens, probiotics, and dietary peptides (Suzuki, 2013). Disruption in paracellular permeability and the epithelium may lead to excessive entry of dietary or microbial antigens, which may be a contributing factor for inflammatory diseases or other diseases (Bischoff et al., 2014).

Direct measurements of intestinal permeability assess intestinal barrier function on a systematic level. It can be measured *in vivo* by analyzing flux rates of inert molecules across the intestinal wall. Most frequently used probes are small sugars and disaccharides, such as mannitol and lactulose (Meddings, 1997; Marshall et al., 2004). The small sized mannitol is thought to freely cross the intestinal barrier, independent of damage, while the larger lactulose can only cross the intestine using the paracellular pathway if the barrier is compromised. The fractional excretion of lactulose to mannitol ratio (LMR) is used as the gold standard for measuring intestinal permeability (Teshima et al., 2012). Indeed, many studies have used LMR to investigate barrier function and found an increased LMR in healthy relatives of CD patients compared to healthy controls (Katz et al., 1989; Buhner et al., 2006; D'Inca et al., 2006; Kevans et al., 2015). However, the LMR test can be laborious to perform, especially in pediatric population. Moreover, there are no standardized protocols (Camilleri et al., 2010; Sequeira et al., 2014), which makes cross-study comparisons difficult. Thus, identifying alternative biomarkers for intestinal permeability is desirable.

Multiple investigations have tested potential indirect biomarkers to assess the integrity of the intestinal barrier (Duerksen et al., 2010; Vorobjova et al., 2017) from serum samples. Among the potential biomarkers, zonula occludens-1(ZO-1) also known as tight junction protein-1 is a promising candidate as an alternative biomarker to measure gut permeability. This is in part because that ZO-1 is a protein that is known to modulate intestinal TJs and is capable of TJ disassembly, which in turn is implicated in the disruption of mucosal permeability (Tripathi et al., 2009). Studies have supported that increased serum levels of ZO-1 can be used as a biomarker of disrupted barrier function in humans (Sapone et al., 2006; Sturgeon and Fasano, 2016). Recent studies in celiac patients and their healthy first-degree relatives (FDRs) suggested that increased intestinal permeability as measured by ZO-1 was associated with indirect evidence of TJ abnormalities (Mishra et al., 2016). ZO-1 has been used in other diseases such as type-1 and 2 diabetes and obesity-related disorders (Sapone et al., 2006), for which it has also been linked to abnormal gut permeability. ZO-1 is also a precursor to haptoglobin-2 and thus belongs to the haptoglobin (HP) family of proteins. In human the HP locus is polymorphic with three haptoglobin types that yield three distinct genotypes/phenotypes (HP1-1, HP1-2, and HP2-2) (Yang et al., 1983) as determined by the *HP1* and *HP2* alleles located on chromosome 16q22 (Langlois and Delanghe, 1996). Individuals who bear the heterozygous HP1-2 or homozygous HP2-2 polymorphisms are ZO-1 producers, whereas those with the homozygous HP1-1 polymorphism are unable to produce ZO-1 (Ajamian et al., 2019). Thus, genotyping of individuals could serve as an additional quality control of ZO-1 testing.

Other biomarkers have been suggested that could capture enterocyte damage and TJ loss, notably by using peptides that are released into circulation such as intestinal fatty acid binding proteins (I-FABP) (Adriaanse et al., 2013). I-FABP is a small protein that is present in mature enterocytes of the small and large intestine and as such, I-FABP presence in the serum is thought to be a measure of enterocyte damage and TJ loss and by extension to barrier function. I-FABP biological role is to transport fatty acids from the apical membrane of the enterocyte to the endoplasmic reticulum where biosynthesis of complex lipids occurs. Circulating basal levels measured in healthy individuals reflect the normal epithelial turnover rate and elevated I-FABP levels have been found in plasma and serum in patients with celiac disease, intestinal ischemia, and systemic inflammatory response syndrome suggesting an abnormal epithelial turnover in these conditions (Pelsers et al., 2005; Derikx et al., 2007, 2009; de Haan et al., 2009).

Although ZO-1 has received considerable attention as a potential biomarker of intestinal barrier dysfunction, there is limited information on the serological measurement of ZO-1 and its relation to other markers of barrier function such as LMR and I-FABP in healthy subjects (Sapone et al., 2006; Duerksen et al., 2010; Linsalata et al., 2018). Our primary aim was to analyze the correlation of serum ZO-1 as measured by the Immunodiagnostik AG kit, with physiologic measures of intestinal permeability as define by the gold standard LMR in a well-described cohort of healthy FDRs of CD.

## Materials and Methods

### Subjects

Healthy FDRs of CD subjects who were between the ages of 6 and 30 years (*n* = 39) were recruited as part of the Crohn's Colitis Canada GEM Project. At the time of recruitment, subjects were screened using a standardized questionnaire to exclude any history or symptoms of IBD or other gastrointestinal diseases, as defined by the clinical subcommittee of the GEM Project as described previously (Turpin et al., 2016). Serum from peripheral blood and urine were collected at the time of recruitment, and stored at −80°C. All study participants and/or their guardians gave written, informed consent and the protocols were approved by Mount Sinai Hospital Research Ethics Committee and at each local recruitment center (see authors in the GEM Project Research Consortium in the acknowledgments).

### Measure of Serum ZO-1 IDK®

Concentrations of ZO-1 in the sera were determined by a commercially available ELISA assay according to the manufacturer's protocol from Immunodiagnostik AG (Bensheim, Germany). Readings were taken using the Synergy H1 plate reader (Biotek, Vermont) at λ = 450 nm against the reference of λ = 620 nm. All study samples and standards were tested in duplicate. Data were analyzed using a four parameters algorithm using GraphPad Prism (GraphPad Prism Software version 7 Inc., CA). This kit is commercialized as a marker of ZO-1. However the kit was recently shown to measure a protein structurally close to ZO-1. Thus, we will refer to ZO-1 mesures of ZO-1 from other kit than the Immunodiagnostik kit and referred to ZO-1 IDK® where referring to protein measured by the Immunodiagnostik kit.

### Measure of Serum I-FABP

Concentrations of I-FABP in the sera were determined by a commercially available ELISA assay according to the manufacturer's protocol from R&D Systems (Minneapolis, USA). Readings were taken using the Synergy H1 plate reader (Biotek, Vermont) at λ = 450 nm against the reference of λ = 540 nm. All study samples and standards were tested in duplicate. Data were analyzed using a 4-parameter algorithm using GraphPad Prism (GraphPad Prism Software version 7 Inc., CA).

### Measurement of LMR

Subjects were required to refrain from ingestion of alcohol, aspirin, and other non-steroidal anti-inflammatory agents for 5 days before probe administration. Study subjects ingested a sugar test solution containing 500 mL of 5 g lactulose and 2 g mannitol before bed. Urine samples were collected the following morning in a container contained 5 mL of thymol solution and returned to the study center. A 5 mL portion of urine was taken and stored at −80°C until analysis. The measurement of the fractional excretion of urinal sugar probes, lactulose and mannitol, was performed using high-pressure liquid chromatography. For each subject, the LMR was calculated as the fractional excretion of lactulose divided by that of mannitol as described previously (Kevans et al., 2015; Turpin et al., 2020).

### Genotyping and Structural Haptoglobin Imputation

As part of a the GEM project (Turpin et al., 2019), DNA was extracted from human peripheral blood mononuclear cells to perform single nucleotide polymorphism genotyping using the HumanCoreEXOME-12v1.1 chip (Illumina, Inc. San Diego, CA) and quality control performed as previously described (Turpin et al., 2019). We imputed haptoglobin structure from 1,561 subjects from the GEM project, HP1-1 or homozygous HP2-2 or and heterozygous HP1-2 (Scheffler et al., 2018) using method described previously (Boettger et al., 2016). The current studies included 36 subject that had haptoglobin structure imputed.

### Statistical Analysis

Statistical analyses were performed with GraphPad Prism software version 7 (GraphPad Prism Software Inc., CA). Quantitative variables were summarized as mean ± Standard Deviation. The median differences between groups were analyzed using Mann-Whitney Student's *T*-test for comparison of groups or Spearman correlation test to address correlation between continuous variable. The cut-off value of LMR > 0.025 was considered as abnormal (Turpin et al., 2019). For all tests, a *P*-value < *0.05* was considered to be statistically significant.

## Results

### Demographic of the Cohort

Demographic details of the participants included (*n* = 39) in this study are shown in Table 1. Age, sex data were recorded, and showed an equal proportion of males to females with a mean age of 21 years. The mean age was 20.9 ± 8.1 and 56.4% were female. We also measured ZO-1 IDK®, LMR, I-FABP, and imputed haptoglobin structure in this cohort as presented in Table 2.

**Table 1:** Demographics and assay sample size of study participants.

	**Subjects (*n* = 39)**
Mean age (yrs ± SD)	20.9 ± 8.1
Males (%)	17.0 (43.6)
LMR assay	39
ZO-1 IDK® assay	39
I-FABP assay	35
Genotyped	36

**Table 2:** Assay ranges of our subjects vs. their reported value in the kits from healthy people.

	**Threshold in GEM (±SD)**	**Recommended threshold for healthy subjects**
LMR assay	0.025 ± 0.01	0.025 ± 0.01
ZO-1 IDK® assay (n/mL)	48 ± 29	34 ± 14
I-FABP assay (pg/mL)	666 ± 5217	1015 ± 455

*LMR threshold is based on reported values in the literature. ZO-1 IDK® and I-FABP are the obtained median values*.

### Measure of Intestinal Permeability by LMR

**Figure 1:**
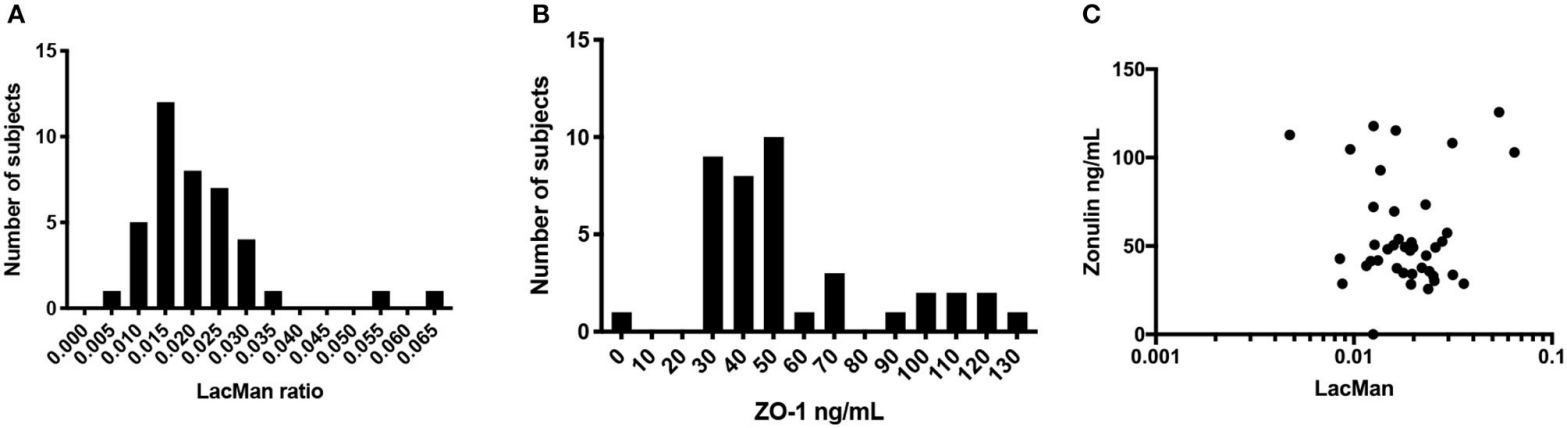
Distribution and correlation of LMR and ZO-1. **(A)** Histogram of LacMan ratio in the study participants. The x axis plots the LacMan ratio and the y axis corresponds to the number of individuals with that measurement. Median LMR is 0.02 ± 0.01 (IQR 0.013–0.025), *n* = 39. **(B)** Histogram of serum ZO-1 IDK® concentration in the study participants. The x axis plots the ZO-1 IDK® concentration and the y axis corresponds to the number of individuals with that measurement. Median ZO-1 is 48 ± 29 ng/mL (IQR 35.1–71.4), *n* = 39. **(C)** Scatter plot of purported serum ZO-1 levels (ng/mL) and LMR. Spearman correlation between the two assays (*r*2 = 0.004, *p* < 0.71), *n* = 39.

LMR ratio is considered as the gold standard to measure gut barrier function (Teshima et al., 2012). As such all other measure presented in Table 2 were directly compared to LMR. The LMR measured in this cohort had a mean value of 0.02 ± 0.01 (IQR 0.013–0.025) (Figure 1A). A cutoff of 0.025 was used based on thresholds previously defined in healthy subjects (Turpin et al., 2020). Of the 39 subjects in this study, 14 had an LMR equal to or above the 0.025 threshold, indicating abnormal intestinal permeability.

### ZO-1 IDK® Does Not Correlate With LMR Marker of Intestinal Permeability

**Figure 2:**
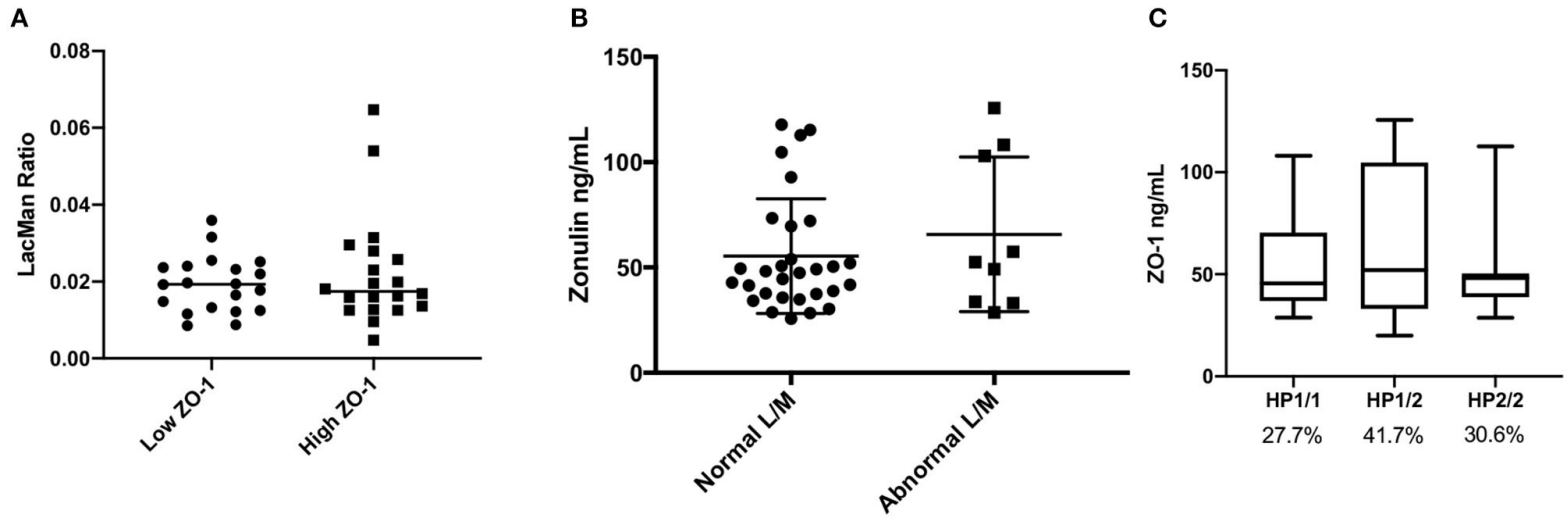
Comparison of LMR, ZO-1 IDK®, and imputed haptoglobin haplotype. **(A)** Beeswarm plot showing the LMR in subjects which are categorized by low and high ZO-1 IDK® concentrations. Threshold level of ZO-1 used was the median, 48 ng/mL. There was no significant difference in LMR values between subjects with ZO-1 IDK® > or < 48 ng/mL groups (Mann Whitney, *p* < 0.82, *n* = 39). **(B)** Beeswarm plot showing the ZO-1 IDK® concentration in our subjects which are categorized by low and high LMR. Threshold level of LMR used was 0.025, as defined in the literature. There was no significant difference in ZO-1 IDK® levels between subjects with LMR > or < 0.025 groups (Mann Whitney, *p* < 0.55, *n* = 39). **(C)** Distribution of ZO-1 IDK® levels according to haptoglobin genotypes. Data are presented as boxplots with Turkey-Whiskers (*n* = 36).

The levels of serum ZO-1 IDK® were estimated in 39 subjects using a commercially available ELISA kit (see section Method). A mean value of serum ZO-1 IDK® in healthy CD FDRs was 56 ± 29 ng/mL (IQR 35.1–71.4) (Figure 1B). We then used a Spearman correlation test to determine whether those with LMR and ZO-1 IDK® levels were correlated (Figure 1C). No significant correlation was found between LMR and ZO-1 IDK® levels (*r*^2^ = 0.004, *P* < 0.71). Next, we dichotomized subjects by low and high levels of ZO-1 IDK®, using the median value of ZO-1 IDK® in the cohort (48 ng/mL). There was no significant difference (*p* < 0.82) in LMR values between subjects with ZO-1 IDK® > or <48 ng/mL groups (Figure 2A). Finally, we dichotomized LMR using the 0.025 threshold and compared with ZO-1 IDK® as a continuous variable (Figure 2B). There was no significant difference in ZO-1 IDK® levels between the subjects with LMR > 0.025 levels and subject with LMR <0.025 (*p* <0.55).

### ZO-1 IDK® Level Does Not Concord With Expected Haptoglobin Phenotype

The kit used to define ZO-1 IDK® in this study has been shown to provide inaccurate measurements in regards to haptoglobin structure (Scheffler et al., 2018). Thus, we estimated its concordance with imputed haptoglobin structure from our genotyping data. Of the 36 subjects with imputed haptoglobin phenotype, 10 (27.7%) were HP1-1, 15 (41.7%) were HP1-2, and 11 (30.6%) were HP2-2. We find no significant differences in the levels of ZO-1 IDK® ELISA levels between the haptoglobin genotype groups (Figure 2C; *p* = 0.729). In addition, subjects with the HP1-1 genotype are expected to have little to no detectable ZO-1 levels (Ajamian et al., 2019), however our results showed that the detect ZO-1 IDK® by the commercial ELISA is present even in the HP1-1 genotype group (Figure 2C).

### Evaluation of I-FABP as a Marker of Intestinal Permeability

**Figure 3:**
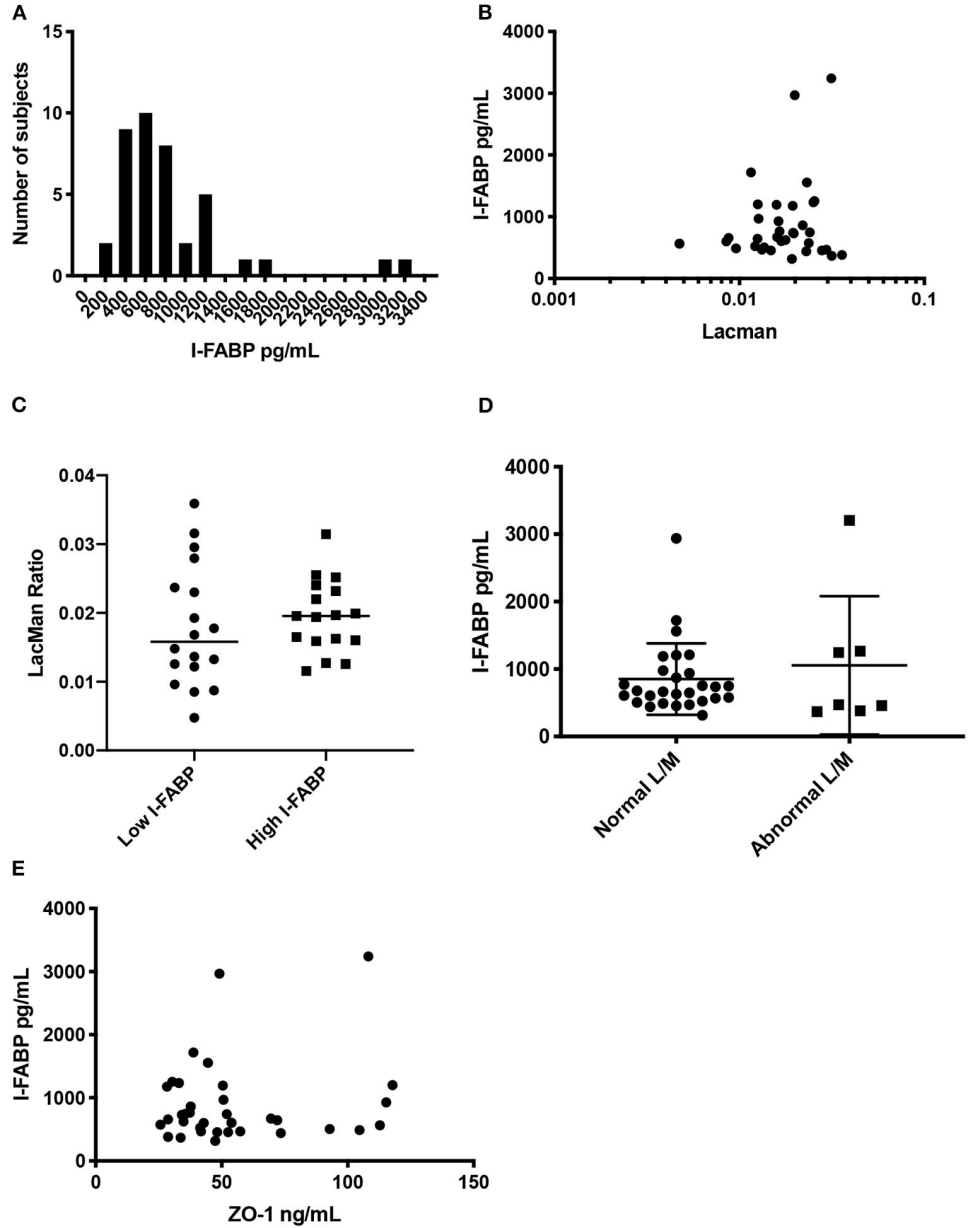
Distribution of I-FABP, and correlation with LMR and ZO-1 IDK®. **(A)** Histogram of serum I-FABP concentration in the study participants. The x axis plots the I-FABP concentration and the y axis corresponds to the number of individuals with that measurement. Median I-FABP is 666 ± 527 pg/ml (IQR 35.1–71.4, *n* = 35). **(B)** Scatter plot of purported serum I-FABP levels (pg/mL) and LMR. **(B)** Spearman correlation between the two assays (*r*2 = 4 × 10-6, *p* < 0.99), *n* = 35. **(C)** Beeswarm plot showing the LMR in our subjects which are categorized by low and high I-FABP concentrations. Threshold level of I-FABP used was the median, 666 pg/mL. There was no significant difference in LMR values between subjects with I-FABP > or < 666 pg/mL groups (Mann Whitney, *p* < 0.64, *n* = 35). **(D)** Beeswarm plot showing the I-FABP concentration in our subjects, which are categorized by low and high LMR. Threshold level of LMR used was 0.025, as defined in the literature. There was no significant difference in I-FABP levels between subjects with LMR > or < 0.025 groups (Mann Whitney, *p* < 0.53, *n* = 35). **(E)** Scatter plot of purported serum I-FABP levels (pg/mL) and ZO-1 IDK® levels (ng/mL). Spearman correlation between the two assays (*r*2 = 0.001, *p* < 0.85), *n* = 35.

Similar to what was described above, we assessed correlations between serum I-FABP and LMR. The level of serum I-FABP was estimated in 35 FDRs using a commercially available ELISA kit (see section Method). The mean value of serum I-FABP was 846 ± 527 pg/mL (IQR 475–959) (Figure 3A). Using a Spearman correlation test (Figure 3B), we found no significant correlation between LMR and I-FABP levels (*r*^2^ = 4 × 10^−6^, *p* < 0.99). Next, we dichotomized subjects by low and high levels of I-FABP, using the median value of 666 pg/mL, and compared the LMR variable in those groups (Figure 3C). There was no significant difference in LMR values between the individuals with I-FABP > or < 666 pg/mL (*p* < 0.64). Similarly subjects with a LMR levels higher or lower than 0.025 had similar level of I-FABP (*p* < 0.53) (Figure 3D). Lastly, we compared ZO-1 IDK® levels with I-FABP levels using a Spearman correlation test (Figure 3E). We found no significant correlation between ZO-1 IDK® and I-FABP levels (*r*^2^ = 001, *p* < 85).

## Discussion

Increasing evidence has suggested that impaired intestinal barrier function may play a role in many diseases. Recently, Keita et al. (2018) demonstrated that barrier dysfunction as measured by biopsies mounted in Ussing chambers, and tight junction protein expression measured using immunofluorescence, is a primary defect in twin pairs that are discordant for CD. Similar studies on T1D and celiac disease (Duerksen et al., 2010; Vorobjova et al., 2017) also found an association between increased intestinal permeability as measured by LMR and increased serum ZO-1 and ZO-1 IDK®. Due to its putative role in reversible TJ disassembly, ZO-1 has emerged as a potential serological marker of barrier function, and integrity of the intestinal barrier mucosa. In this study we focused on the evaluation of two serum markers of the gastrointestinal barrier function as alternative method to LMR in a well-characterized cohort of healthy FDRs of CD. We previously established that the intestinal permeability was abnormal in a larger proportion of CD FDR, as evidenced by higher LMR compared to reported proportion observed in individuals from the general population (Kevans et al., 2015). We used a subset of this cohort with a range of normal to abnormal LMR and assessed their circulating ZO-1 IDK® and I-FABP.

The mean measured ZO-1 IDK® was of 56 ± 29 ng/mL in this cohort suggesting that the majority of the subject had abnormal ZO-1 levels as defined by the kits recommended level of 34 ± 14 ng/mL (Fisher's exact test *p* < 0.037). However ZO-1 IDK® level was in line with reported value of ZO-1 observed in healthy individuals in another study (Linsalata et al., 2018). It remains unclear what is the optimized ZO-1 IDK® threshold should be used to estimate abnormal ZO-1 IDK® level. In contrast to the mentioned studies (Sapone et al., 2006; Duerksen et al., 2010), we found no correlation between the physiologic measurement of intestinal permeability estimated by LMR and estimated by serum ZO-1 IDK®. However the absence of correlation was in agreement with Linsalata et al. (2018) that also found no correlation between LMR and ZO-1 IDK®. Our data suggest that the physiologic measurement of intestinal permeability may not correlate with changes in TJ structure. Alternatively, other factors affecting permeability reflected by LMR may occur independently of ZO-1. Indeed, there may not be significant loss of ZO-1 absolute quantity in the epithelium, but rather a redistribution and localization of TJ proteins with a similar absolute quantity of ZO-1. The absence of correlation between LMR and ZO-1 IDK® may also be explained by the ELISA kit use to evaluate ZO-1 expression in serum.

To date there are several published reports analyzing ZO-1 as a marker of intestinal permeability (Teshima et al., 2012; Adriaanse et al., 2013; Vojdani et al., 2017; Vorobjova et al., 2017; Linsalata et al., 2018). Of these studies, eight used the Immunodiagnostik (IDK) ELISA kit, while the remaining used alternate ELISA kits or made them in house. The studies that utilized in-house made ZO-1 ELISA plates (*n* = 5), revealed consistent results that increased permeability measured by LMR is associated with increased ZO-1. Of those that used the IDK ELISA kit, three of them found an increase in peripheral ZO-1 IDK® correlated with celiac patients, vs. healthy controls (Vojdani et al., 2017; Vorobjova et al., 2017; Linsalata et al., 2018), while two of them discovered that the kits antigen does not directly bind to ZO-1 (Scheffler et al., 2018; Ajamian et al., 2019). Overall, there is conflicting data regarding the use of commercial ZO-1 IDK® ELISA kits to measure gut barrier function and whether the kit is detecting other proteins. In addition we expected that subjects with the HP1-1 genotype to have very low levels of ZO-1 (Ajamian et al., 2019). However, we find the ZO-1 IDK® concentrations measured in serum using the IDK ELISA kit were similar to those found in the HP1-2 and HP2-2 carriers. Thus, the IDK ELISA kit did not reflect the expected haptoglobin genotype distribution in our cohort. These results are also supported by previous studies that have determined haptoglobin phenotype distribution and ZO-1 levels using IDK kits (Scheffler et al., 2018; Ajamian et al., 2019). Overall, the conflicting evidence, and lack of concordance with expected haploglobin phenotype suggest that the IDK commercial assays may not be detecting the protein as advertised, and thus by extension do not measure gut permeability.

Data are limited in measured difference of I-FABP and chronic diseases. Although studies have suggested that I-FABP may reflect intestinal damage, and thus impaired barrier function, we showed no correlations of I-FABP with LMR or serum ZO-1. In contrary, Linsalata et al. (2018) performed a similar test on celiac disease patients and healthy controls and found a positive correlation between LMR and I-FABP. The I-FABP levels was lower than the kit recommended level of 1015 ± 455 pg/ml (Threshold used to classify healthy individuals), and also lower than a described “healthy” levels, although this study used a different manufactured kit (Linsalata et al., 2018). It remains possible that I-FABP may still be useful as a biomarker to identify localized changes in acute intestinal damage (Grootjans et al., 2010; Galipeau and Verdu, 2016), since they are released into circulation upon enterocyte damage and cleared rapidly (half-life of 11 min). Indeed, I-FABP is predominantly expressed in the jejunum (Galipeau and Verdu, 2016), and thus I-FABP may not show differences if abnormal intestinal permeability is occurring at the other sites of the gastrointestinal tract. This suggests that different FABP may provide more information on disease location but would require one to measure multiple types of FABP.

The data described here showed that ZO-1 IDK® and I-FABP are not reliable markers of gut permeability as defined by LMR. It is important to note that our study was not set up to address the specificity of the ELISA kit. While we used serum from subject that exhibited high and low LMR values, the small size of cohort may be a limiting factor. It remains to be shown if other measures of ZO-1 or TJs proteins would correlate with LMR. The correlation between established biomarkers of gut permeability with functional permeability measure by LMR has not been well-studied. Nonetheless, our results provide a starting point for future studies to better define alternative biomarkers of intestinal permeability. Further studies are needed to identify and validate alternative targets of intestinal permeability and barrier function. Additionally, studies are necessary to establish the primary antigen detected by commercially available ZO-1 ELISA kits in order to understand to functional relevant of the IDK ELISA kits and its relation to gut barrier function.

## Data Availability Statement

The datasets presented in this article are not readily available because sharing the individual-level genomics data might compromise the privacy of individual participants. Requests to access the datasets should be directed to http://www.gemproject.ca/data-access/.

## Ethics Statement

The studies involving human participants were reviewed and approved by Mount Sinai Hospital Research Ethics Committee and at each local recruitment center. Written informed consent to participate in this study was provided by the participants' legal guardian/next of kin.

## Authors in the CCC GEM Project Research Consortium

The CCC GEM Project Research Consortium is composed of: Maria Abreu, Paul Beck, Charles Bernstein, Kenneth Croitoru, Leo Dieleman, Brian Feagan, Anne Griffiths, David Guttman, Kevan Jacobson, Gilaad Kaplan, Denis O. Krause^*^, Karen Madsen, John Marshall, Paul Moayyedi, Mark Ropeleski, Ernest Seidman^*^, Mark Silverberg, Scott Snapper, Andy Stadnyk, Hillary Steinhart, Michael Surette, Dan Turner, Thomas Walters, Bruce Vallance, Guy Aumais, Alain Bitton, Maria Cino, Jeff Critch, Lee Denson, Colette Deslandres, Wael El-Matary, Hans Herfarth, Peter Higgins, Hien Huynh, Jeff Hyams, David Mack, Jerry McGrath, Anthony Otley, and Remo Panancionne. The CCC GEM Project recruitment site directors include Maria Abreu, Guy Aumais, Robert Baldassano, Charles Bernstein, Maria Cino, Lee Denson, Colette Deslandres, Wael El-Matary, Anne M. Griffiths, Charlotte Hedin, Hans Herfarth, Peter Higgins, Seamus Hussey, Hien Hyams, Kevan Jacobson, David Keljo, David Kevans, Charlie Lees, David Mack, John Marshall, Jerry McGrath, Sanjay Murthy, Anthony Otley, Remo Panaccione, Nimisha Parekh, Sophie Plamondon, Graham Radford-Smith, Mark Ropeleski, Joel Rosh, David Rubin, Michael Schultz, Ernest Seidman^*^, Corey Siegel, Scott Snapper, Hillary Steinhart, and Dan Turner. (^*^ deceased).

## Author Contributions

NP, WT, OE-G, and MS: analyzed the data. The CCC GEM Project Research Consortium and KC: recruited patient. NP, WT, and KC: wrote the manuscript. All authors contributed to the article and approved the submitted version.

## Conflict of Interest

The authors declare that the research was conducted in the absence of any commercial or financial relationships that could be construed as a potential conflict of interest.
